# Comment on ‘Dairy, calcium, vitamin D, and ovarian cancer risk in African-American women’

**DOI:** 10.1038/s41416-018-0166-y

**Published:** 2018-07-02

**Authors:** Constance Hilliard

**Affiliations:** University of North Texas, Denton, TX 76203 USA

**Keywords:** Ovarian cancer, Cancer epidemiology

In September 2016, an article by Qin et al.^1^ in the *British Journal of Cancer* advised Black females to increase their calcium intake as a means of reducing their susceptibility to the disease. Due to the severity of the cancer among this population and the lack of therapeutic progress in combating it, this research was disseminated widely throughout the United States and given special attention in the African-American media. However, my own work as an evolutionary African historian relies on findings at the intersection of population genetics and oncology. It concludes that Black women should not be encouraged to consume more dietary calcium, as this will increase their risk of ovarian cancer. The reason is that this population carries an ethnic-specific variant of the TRPV6 calcium ion channel (referred to as TRPV6a),^2^ which is hypersensitive to carcinogenic-triggering free calcium ions. Furthermore, a growing body of research points to the over-consumption of calcium as triggering metastatic cancers.^3,4^ While it is easy to overlook African-Americans because this group does not “overconsume” the mineral by American standards, their intake is two to four times more than their African ancestors, who carry the same variant.

Genetic researchers have now begun to categorise ovarian cancer along with metastatic prostate cancer and triple negative breast cancer as TRPV6-expressing malignancies, because of the telltale proliferation of TRPV6 mRNA in the metastasising tissue of these diseases.^5^ My work applies an Ecological Model to these findings. In so doing, it explains, for instance, why the descendants of Africans in America have twice the mortality rate of Whites in confronting these aggressive cancers. A genetic variant that benefited their ancestors in the low-calcium regions of West Africa is proving to be oncologically maladaptive now that these descendants find themselves relocated to the dairy-rich food cultures in the West.

In fact, the medical community had for decades noted what they termed a “paradox” involving African-American women. This group exhibited strong bones and the lowest rate of osteoporosis of any ethnic group in the United States, while appearing calcium-deficient by USDA dietary standards on account of having the lactase non-persistent trait. What we now know to be the case is that the African TRPV6a variant is far more absorbent of dietary calcium than the non-African/European TRPV6b allele. My research traced this genetic trait back to the Niger-Kordofanian ancestors of this group, who maintained strong skeletal health on 200–400 mg/calcium/day, while inhabiting a zone of West Africa infested by the tsetse fly glossina, which made dairy pastoralism unsustainable.^6^ But such beneficial alleles are maladaptive and possibly even carcinogenic in the 1000–1200 mg calcium/day dairy food culture of the United States, because the TRPV6a calcium ion channel is absorbing 25% more calcium and the African TRPV5 gene is retaining rather than expelling calcium in the urine (in contrast to the European TRPV5 variant).^7^

Nonetheless, the 2016 article performed an important service. It represented an effort to unravel the unusually high rate of ovarian cancer among an understudied community, that of African-American females. However, the design of the study was flawed because its “one size fits all” methodology perhaps universalised European measures, applying those values to all human populations. The article, for instance, began with two claims, which given rapid developments in the field are no longer considered valid. It is that “African-American women…[are] at risk for calcium and vitamin D deficiency.”

This letter will not take up the vitamin D issue. But suffice it to say that the most recent findings have shown that for Caucasians, blood levels of 25-hydroxyvitamin D are a valid indicator of their bioavailable vitamin. However, medical researchers now say that Blacks have been mistakenly diagnosed as being vitamin D deficient.^8^ No basis exists for claiming that African-American women are deficient in calcium but for the fact that they consume less than Whites. The lower risk of osteoporosis belies the calcium deficiency argument relating to Black females. But perhaps of greater importance is the fact that the preoccupation with calcium supplementation as a means of improving bone health for females in Western nations may have blinded researchers to the possibility that certain non-European ethnic populations can be harmed by calcium intake beyond their ethnic biology’s setpoint.

The article’s statistical analyses correlating ovarian cancer and dairy/calcium consumption also reflect the study’s design flaws. Because the data does not show what it purports to show, the authors’ conclusions cannot be supported by the data provided as explained below:

The article states: “In this population-based ovarian cancer study of AA women, the positive association between the total dairy intake and ovarian cancer risk seemed to be attributable to the consumption of whole milk.” and “… we found lactose intake increased ovarian cancer risk in AAs.”

However, the authors have not identified in either of these conclusions specific characteristics of ovarian cancer in African-American women, who are an admixed population of approximately 75% Niger-Kordofanian/24% Northern European, 1% Native American ancestry. To the contrary, the researchers have (perhaps inadvertently) selected out of a mixed-race population, the segment of “Blacks” that has between 50 and 75% Northern European genetic ancestry. These are the women who drink milk and consume lactose. Those with larger mixtures of Niger-Kordofanian ancestry are lactase non-persistent, lack the genetic variant required to hydrolyze the lactose in milk and are represented on Table 2 in the manuscript as the 72.7% “non-consumers.” However, several studies have already been done linking whole milk and lactose to ovarian cancer in White women. While it would be safe to reinforce that message, this milk/lactose issue is not causing Black women’s high susceptibility to ovarian cancer, again since the 72.7% of these cancer patients as shown in the table do not drink milk.

The article states: “Calcium intake was associated with a decreased risk of ovarian cancer (OR = 0.51, 95 CI%: 0.30–0.86)”.

However, Table 3 in the article (replicated as Table 1) does not show calcium intake associated with a decreased risk of ovarian cancer in African-American women. Rather, it shows that in the transition from the 70+% lactose intolerant majority of Black women to the 30% lactose tolerant minority with predominantly European ancestry, fewer will be in the highest quartile of calcium consumption. The danger here is that a stratification bias will show no Black women with ovarian cancer at the highest levels of calcium consumption. Calcium will not have eliminated their risk of contracting the disease. Rather, their lactose non-persistence (lactose intolerance) and hypersensitivity to calcium will cap both their calcium consumption and their ovarian cancer incidence at a lower end of the calcium intake scale than White women.

**Table 1:** Association between intakes of calcium, vitamin D, and lactose with ovarian cancer risk in AACES

Total calcium (mg/day)	Cases (*n* = 490)		Controls (*n* = 656)		Model 1		Model 2	
	*n*	%	*n*	%	OR	95% CI	OR	95% CI
Q1 (≤478.6)	298	26.0	164	25.0	1.00	Ref	1.00	Ref
Q2 (478.7–784.1)	306	26.7	164	25.0	1.00	0.72, 1.39	0.89	0.61, 1.31
Q3 (784.2–1233.6)	272	23.7	164	25.0	0.70	0.48, 1.00	0.62	0.39, 0.96
Q4 (≥1233.7)	270	23.6	164	25.0	0.63	0.42, 0.94	0.51	0.30, 0.86

*AACES* African American Cancer Epidemiology Study, *CI* confidence interval, *IU* international unit, *OR* odds ratio

It is altogether possible that dietary calcium standards that work best for females of Northern European ancestry in protecting them from osteoporosis, might in fact trigger metastatic cancers in women of African ancestry. It will take further study in complementary fields of human biology, medicine and genetics to answer the question of ethnic differences in calcium homeostasis, not merely as regards Blacks, but for other non-European populations as well. Until such definitive studies are done, no new recommendations should be made for changing the calcium intake of African-American women with or without ovarian cancer.
